# Status of North American Graduate Programs in Periodontics Providing Laser Education and Clinical Training: A Cross-Sectional Survey

**DOI:** 10.3390/dj10070119

**Published:** 2022-07-01

**Authors:** Andre Paes Batista da SIlva, Uma M. Irfan, Elena Furman, T. Roma Jasinevicius

**Affiliations:** 1 Department of Periodontics, Health Education Campus, Case Western Reserve University, Dental Clinic/2nd Fl–240, 9601 Chester Avenue, Cleveland, OH 44106, USA; exf19@case.edu; 2 School of Dental Medicine, Case Western Reserve University, Cleveland, OH 44106, USA; uxp3@case.edu; 3 Department of Comprehensive Care, Health Education Campus, Case Western Reserve University, Dental Clinic Rm 396A, 9601 Chester Avenue, Cleveland, OH 44106, USA; trj2@case.edu

**Keywords:** graduate periodontics program, laser education, laser training, laser use

## Abstract

The aim of this cross-sectional study was to evaluate the status of laser use and training in the U.S. and Canadian graduate periodontology programs. A survey questionnaire was sent electronically to 55 periodontology program directors in North America. The questions focused on laser implementation, types of lasers used, for which procedures lasers were used, and level of education/clinical training provided to residents. Data were analyzed using descriptive statistics and Fisher’s exact test. Twenty-two directors responded (40%). Most programs (86%) used lasers and 89% used a diode laser. Laser treatment was the most used for periimplantitis (84%). Fifteen programs (79%) provided didactic and clinical training, with 47% programs giving 4–12 h of didactic training. In 53% of programs, residents completed 4 to10 procedures. Only 29% of programs had residents who had received a certification in lasers, with most (80%) programs requiring between 1–9 cases for certification. Of the participants not providing laser training, the major barrier was indicated as being “expense”, with 68.7% reporting plans to implement laser education. Conclusions: Most graduate periodontics programs were providing laser training and treatment. There was great variability regarding the training methods, specifically in number of dedicated laser courses, time allocated for laser training, and prerequisites for laser certification.

## 1. Introduction

The use of lasers by periodontists for the treatment of periodontitis, and especially for peri-implantitis, has been increasing in recent years. Dental lasers have several periodontal applications, including calculus removal; soft tissue excision, incision and ablation; bacterial reduction or decontamination of root and implant surfaces; photobiomodulation; de-epitheliazation; hemostasis; and alveolar bone removal [1,2]. When compared with conventional/mechanical methods, laser treatments have demonstrated improved subgingival calculus removal [3], decreased bacterial load at the treated site [4,5,6,7], and improved removal of periodontal pocket epithelium [8,9]. In dental implant therapy, it has been suggested that laser therapy is the state of the art compared with conventional techniques [10]. In a recent systematic review, lasers were promising in encouraging bone fill around implants; however, further studies were needed with proper statistical analysis of the bone level changes [11]. In addition to monotherapy laser use, two systematic reviews included studies in which lasers were used as an adjunctive to the surgical and non-surgical treatment of perimplantitis [11,12].

In dentistry, lasers can be grouped according to their wavelength, the affected tissue (soft tissue and hard tissue), and the medium of laser used (solid and gas). The four laser types used in dentistry are diodes, CO_2_, erbium, and NdYag [1]. The erbium laser wavelength is highly absorbed by hydroxyapatite and can be used for bone removal, whereas diode and the neodymium yttrium aluminum garnet lasers (Nd: YAG) are highly absorbed by hemoglobin and pigmentation; therefore, they are used when coagulation is desirable, for the removal of gingival pigmentation and for the reduction of black-pigmented bacteria. The CO_2_ laser, because of its high affinity for water, induces rapid soft tissue elimination and hemostasis with a very shallow depth of penetration. The additional advantages of laser use in periodontal therapy include decreased pain levels, hence decreased anesthetics; lower risk of bacteremia; excellent wound healing; improved bleeding control; no need for sutures; use of fewer instruments and materials; and the ability to remove both hard and soft tissues [1,2]. Some of the aforementioned advantages are critical when treating medically compromised patients. Clearly, full knowledge on the use of a specific laser (wavelength and settings), interactions with oral/periodontal tissues, and adhering to each laser’s prescribed protocols are essential to achieve the desired goals. Some disadvantages when compared with conventional periodontal treatment include the high cost of most devices; laser wavelengths have different properties, hence the need of further education; and adopting requisite safety measures for laser use [1,2].

The results of periodontal laser treatment are similar to more invasive surgical procedures, such as the treatment of periodontitis and peri-implantitis, gingivectomy, frenectomy, operculectomy, removal of aphthous ulcerations, and the exposure of impacted teeth and implants [1], but often with a better patient acceptability, in part, due to less post-operative discomfort. Clearly, with increased use and patient demand, laser training should be a component of graduate periodontics education. Residents who have received laser training should be more prepared to recognize appropriate conditions for laser use, and consequently provide safe and optimal patient care. However, only one recent study has assessed the degree to which periodontics residencies have implemented laser education into their curriculum [13].

There seems to be an emphasis on promoting lasers as a marketing device. Institutions should provide proper education on the use of lasers. Therefore, the aim of this study is to evaluate the status of education in lasers in graduate programs in periodontology. The three main objectives of this study are the following: (1) to investigate if and how the North American periodontics residency programs are training periodontics residents to use lasers, (2) to determine which type and how lasers are being used, and (3) to determine the interest and plans for laser training in periodontics programs.

## 2. Methods

### 2.1. Study Design

This was a cross-sectional survey conducted to elicit information on laser training and education in graduate periodontics programs in the U.S. and Canada. The research protocol for this study was approved by the Case Western Reserve University (CWRU) Institutional Review Board as being exempt from oversight (IRB-2020-1632). All of the methods were carried out in accordance with CWRU IRB guidelines and regulations.

### 2.2. Study Sample

This was a non-probability purposive sample of Graduate Periodontics Program Directors in the regions of the U.S. and Canada. The sampling frame included all (N = 55) graduate periodontics program directors in the six selected regions of the U.S. and Canada. The resulting study sample included 22 program directors’ responses with a 40% response rate, which was similar to other surveys in qualitative dental research.

### 2.3. Survey

In 2020 (December) and 2021, a cover letter with a link to a short survey was sent electronically to 55 advanced periodontology graduate program directors in the U.S and Canada. The survey was placed on the Google Forms website; the link to participate in the survey was included in the cover letter that was e-mailed periodontology program directors listed in the American Academy of Periodontology (AAP) website. In addition, a cover letter explained the purpose of the study, described how the data would be aggregated and used, and noted that all responses would be anonymous. The invitation to participate in the survey was sent four times by e-mail at 30-day intervals.

The question items had categorical/response options. A type of jump logic was used to skip to next item for certain questions, based on the recipient’s response. In the present study, the survey questions (although slightly modified) modelled those in other studies assessing laser use [14,15,16]. The survey question format with categorical choices was designed to allow for a quick response. To ascertain if there were regional differences, the first question asked the program directors to identify in which of the following regions they were located: West, Midwest, Southwest, Northeast, Southeast, or Canadian institutions.

### 2.4. Statistical Analysis

Statistical analysis was done using EpiInfo version 7.2.1 with descriptive statistics and Fisher’s exact test to determine if there were differences in laser usage, types of lasers used, and the types of training among the graduate periodontics programs in the U.S. and Canada. Statistical significance was set at *p* < 0.05.

## 3. Results

We received 22 responses from the 55 advanced periodontology programs (response rate of 40%). Not all questions were required to be answered by all addressees if they were not applicable to a particular program.

### 3.1. Periodontology Advanced Program Characteristics and Their Use of Lasers

**Figure 1:**
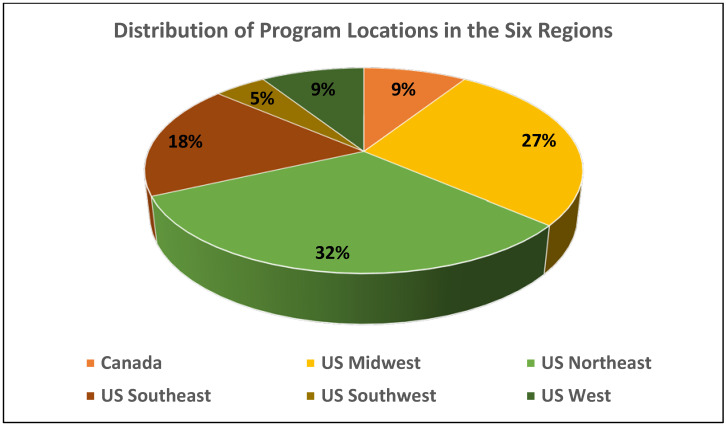
Distribution of the participating program locations in the six regions.

To investigate regional differences, the program location was separated into six regions. The majority of respondent programs were from the Northeast (32%) and Midwest (27%) (Figure 1). The majority (64%) of programs had between 7–12 residents enrolled. Most of the respondent programs, 19 out of 22, were using lasers (86%) for education and training in a clinical setting. Some of the programs were using more than one type of laser. Of the 19 programs that used lasers, the majority used diodes (17, 89%) followed by NdYag (10, 53%). Only seven (37%) programs had erbium lasers and they were mostly located in the northeast U.S. (*p* < 0.05) (Table 1 and Supplemental Table S1). Approximately half of the respondents used lasers for clinical treatment and research, and the other half for clinical treatment only (52%). Laser treatment was usually provided by both the periodontics faculty and residents (63%) (Table 1).

Lasers were mostly used for periimplantitis treatment (84%), followed by frenectomy (79%), gingivectomy (68%), and periodontitis treatment (58%) (Table 2). The periodontal procedure of frenectomy was significantly (*p* < 0.05) associated with the use of diode lasers in the graduate programs compared with other periodontal procedures (Table S2).

### 3.2. Laser Education

All of the respondent programs that used lasers (19) were providing laser training. Fifteen (79%) programs out of nineteen provided didactic and clinical training. Four programs provided either clinical (2) or didactic training (2). Nine (60%) programs had a specific didactic course for laser training. Out of the 15 programs that provided didactic training, only two programs (13%) had less than 4 h of didactic training, seven (47%) programs had between 4–12 h, and six (40%) programs had over 12 h of training, and they were located mostly in the northeast of the U.S. (Table 3 and Table S3).

Of the 17 programs in which residents were performing clinical procedures, nine (53%) programs had residents completing between 4–10 procedures and 4 (24%) programs had residents ompleting over 11 procedures. Of the 19 programs that provided training to residents, 11 programs had the periodontology department faculty providing the training. No more than two faculties from their periodontology departments provided laser training, with the majority of programs being trained by one faculty (64%) (Table 3). Of the 17 respondent programs that provided training, only five (29%) provided an option to receive laser certification. Of the five programs, four (80%) required between one to nine cases to be completed for certification and only one program had a minimum requirement of completion of 10–15 cases to be completed for certification (Table 4).

### 3.3. Periodontology Program Directors’ Interest and Plans in Providing Laser Education

The major barrier preventing the incorporation of laser technology into the periodontics residency/advanced program was indicated as “expense” by the survey participants. While addressing the participants’ interest or plans for the future implementation of clinical laser training in the periodontics program, 68.7% participants said that they had plans to do so in the next three years, while the remaining participants (33.3%) reported that they had interest in clinical laser training but did not have any proposed plans for implementation. About 50% of respondents considered the “diode” and 50% considered “NdYag” as their preferred type of laser to implement training in their program.

The general observations and comments (verbatim) made by the respondents on the topic of laser training in the periodontics program is as follows: “Financial resources to purchase laser is unavailable, while companies were not willing to partner with clinicians”; “Lasers are rarely used due to lack of evidence and availability of other alternatives”; “Major reason for not implementing NdYag lasers is expense along with lack of evidence to justify the expense”; “It is not a common treatment option in the clinic and is rarely used”; “Trying to set up certification, but not been able to implement”; “There are 3 LANAP NdYag (Millenium Dental Technologies) instructors on the faculty”; and “Laser applications are limited to those supported by the ADA Council on Scientific Affairs. The NdYag LANAP and the intramuscular use of the NdYag laser are particularly avoided”.

## 4. Discussion

The Commission on Dental Accreditation (CODA) standard 2.8 e. mandates that dental institutions at the predoctoral level must incorporate new technologies in order to “support the dental education program curriculum” [17]. In 2015, dental school deans were surveyed to determine which of 12 new technologies were being implemented in their predoctoral curriculum. Soft tissue and hard tissue lasers were two of the twelve, which included CAD/CAM, cone beam radiography, rotary endodontics, and warm gutta percha, among others. Of the schools responding, lasers were used for the treatment of soft tissues (39%) and hard tissue (12%) in their clinics [18]. Clearly, it follows that the affiliated U.S. and Canadian graduate programs in periodontics would be at the forefront of new technology implementation and assessment, specifically for acquiring and evaluating lasers.

With the rapid increase in the adaptation of laser treatment by periodontists in clinical practice, universities have started assessing student knowledge of lasers [15,19], as well as implementation of laser training in graduate programs [13,14]. According to a survey sent in 2017 to U.S. and Canadian advanced programs in periodontics, 76% of those responding reported that lasers were used in their programs and 37% were used for research purposes [13]. Although it is not possible to do a scientific comparison to the Hamada et al. study [13], as the sample sizes of the mentioned study and current study are small (49% and 40%, respectively) and the names of programs responding are not identified in either study. Four years later, in the present study, the numbers suggest there has been an increase in graduate periodontal programs incorporating laser technology, with 86% of the respondents reporting laser utilization and training and 47% reporting laser use for research.

Not surprisingly, in the current study, the type of lasers used most often were diode (89%) followed by NdYag (52.6%). While Hamada et al.’s earlier study also found diode lasers were the most often used, the usage was lower (65%), and the CO_2_ lasers were second at 39%, with both NdYag and Erbium tied for last place (26.1%) [13]. The diode lasers are used for many common soft tissue procedures including crown lengthening, gingivoplasty, removal of inflamed soft tissue, frenectomies, biostimulation, disinfection of periodontal pockets, and treatment of perimplantitis [2,20,21]. Not only can the diode laser be used for a number of periodontal treatments, part of its popularity could be accredited to its relatively low cost and user-friendliness when compared with other laser wavelengths. In addition, the diode laser has been reported to be an excellent choice for frenectomies, because of the reduced pain levels and faster healing time [22]. Interestingly, in the present study, laser treatment for frenectomy procedures was significantly associated with programs using diode lasers. Similarly, the NdYag laser is used in a variety of periodontal procedures, as well as in the treatment of periodontitis and periimplantitis, including a laser-assisted new attachment procedure (LANAP) and laser assisted peri-implantitis protocol (LAPIP) [23]. In addition, Erbium and CO_2_ lasers are utilized in a variety of periodontal therapies including periimplantitis treatment [24,25].

In the present study, lasers were mostly being used for the treatment of periimplantitis, followed by frenectomies, gingivectomies, and treatment of periodontitis. This was in agreement with the type of lasers most frequently used in the periodontics programs and the modality of treatment that they are known to be effective. In recent systematic reviews, lasers were considered a promising modality for the treatment of periimplantitis [11,12]. Although erbium lasers are becoming popular for the treatment of periimplantitis [11,12], they are still mostly used for hard tissue procedures. Therefore, this may explain its lower popularity in comparison with diodes and NdYag when being used in periodontics programs where mainly soft tissue procedures are performed. A study [13] surveying periodontal graduate programs in 2017 using similar methodology and aims reported that the diode laser was mostly used in their clinics, and that soft tissue procedures such as frenectomies, gingivectomies, and canine exposures were treated using lasers. However, in the earlier study, the second most used laser was CO_2_ [13] as opposed to NdYag, as used in our study. This difference may be partially explained by the increased use of NdYag in recent years with the LANAP and LAPIP protocols for treating periodontitis and periimplantitis cases, as our study indicated that the treatment of periimplantitis was the most common laser treatment modality.

This study indicated that despite all programs that used lasers providing laser training, the amount and level of laser education and clinical practice residents receive greatly varied among programs. A few of the programs provided a specific didactic course, and this is in agreement with a previous study that reported that most programs provide a formal didactic course [13]. Approximately half of the programs had between 4–12 h courses and other programs had over 12 h courses, and they were located mostly in the northeast of the U.S. The Academy of Laser Dentistry (ALD) offers a dental laser standard level course with a minimum of 12 h to obtain certification [26]. Accordingly, it seems that only a small proportion of the programs were providing laser didactic courses with a minimum standard length of time to qualify for certification. In our study, of the few programs that provided certification, most required that residents completed five or more clinical cases to obtain certification. Interestingly, a couple programs required only between one to four clinical cases for certification. These low clinical case numbers may, in part, be related to the types of certifications (institution versus commercial) and the wording of the questionnaire’s categorical response. As previously reported, certification in lasers in orthodontics advanced programs was also low, with few orthodontic programs providing laser certification from ALD, their own institutions, or from other continuing education providers in dentistry [14]. The great variability regarding the requirements for certification among the programs may be a reflection of the many laser certifications available, as most laser manufacturers provide their own training [27]. Laser dentistry certification programs offer both didactic and clinical training to help clinicians achieve competency in laser dentistry [26,28]. Because of the many lasers wavelengths properties and modalities of delivery, one would expect graduate programs to have at least one faculty member with advanced training at “the instructor level” to teach residents at a level that would make them eligible for certification. In our study, a majority of the programs that were providing laser training did indeed have at least one periodontics faculty responsible for the laser instruction/curriculum; however, the type of training/experience they had was not requested.

Expense was considered to be the main factor that prevented laser use and training for some programs. A previous study on laser education in periodontics [13] and orthodontics advanced training [14] reported similar results. The cost of dental lasers varies; certain lasers cost a few thousand dollars and cost others over USD 100,000 [28]. However, as the number of manufacturers has increased, the cost of lasers has decreased. More recently, there has been an increase in laser systems with a dual and triple wavelength, which allows for a broader variety of treatments. All of the programs reported interest in implementing lasers in their programs with only one program reporting that they did not have any plans. In the current study, only one program mentioned lack of evidence to justify cost as a barrier to implement lasers in their programs, whereas in the 2017 study [13], four programs (57%) indicated that as a rationale. In 2018, an expert panel of the American Academy of Periodontology, in a best evidence consensus report, stated that the evidence for laser treatment was “insufficient to support broad conclusions and/or clinical practice guidelines” [29]. In a press release (2018) [30], the AAP president pointed out that “The latest findings are not meant to insinuate that laser usage is more or less beneficial over traditional periodontal therapy or to imply an Academy position on its use in patient care. They simply demonstrate that, in the absence of a critical mass of evidence, all practitioners must rely on their judgement and expert training to make the best possible treatment decisions”.

There were limitations to this study. It is possible that not all program directors or chairs in all U.S. and Canada programs were contacted, if their AAP directory website contact information was outdated (i.e., they were no longer at the institution) or was incorrect. Another limitation is the low response rate of 40%, which could be, in part, as a result of the aforementioned issue. Another limitation is the possible bias respondents might have regarding the training and use of lasers. The responding programs could have been the ones that were currently acquiring experience in laser education. Nevertheless, despite the sample size, this study detected interesting and informative characteristics that portray laser clinical practice and education in periodontics residencies programs in North America.

Just as the CODA periodontology standard regarding didactic instruction and clinical training in dental implants includes the evaluation and management of peri-implant tissues, the management of implant complications and peri-implant diseases, and the maintenance of dental implants [31], the standard should also apply to laser therapies directed at peri-implant tissues. The current study found that 84% of respondents were using lasers for periimplantitis. The curriculum of programs educating about lasers, especially those providing certification in laser use, should be more comparable not only in the number of hours dedicated to didactic training, but also in the requisite clinical experiences of the various treatments. The authors call on the leaders in periodontal education to convene focus groups of educators, practitioners, and researchers to continue this assessment about what should be taught, at what level should it be taught, and what level of training the faculty need to teach, and to make recommendations for advanced periodontal programs.

## 5. Conclusions

Most programs in the U.S. and Canada use lasers for periodontal treatments and most offer education and training in lasers to their residents. There is, however, great variability among the programs in the level of training offered at institutions; not all offer a course specific to laser treatment, and the time allocated for course instruction and clinical training varies, as do the requirements for certification. Clearly, standardization of the training levels that the faculty receive, as well as the curriculum hours and clinical experiences that residents receive, would lead to more consistent laser treatment outcomes. It is imperative that leaders in periodontal education, especially those trained in laser technology, work together to establish a laser curriculum “toolkit” for periodontal programs.

## Figures and Tables

**Table 1:** General information related to use of lasers by periodontal professionals in the U.S. and Canada.

General Information	Number (%)
Number of residents in the graduate periodontics programs (n = 22):	
1–3	1 (4.6)
4–6	3 (13.6)
7–12	14 (63.6)
Over 13	4 (18.2)
Lasers used in the periodontics graduate program (n = 22):	
Yes	19 (86.4)
No	3 (13.6)
Types of lasers used: *	
Diode	17 (89.5)
NdYag	10 (52.6)
Erbium	7 (36.8)
CO_2_	7 (36.8)
Lasers used for:	
Clinical Treatment	10 (52.6)
Clinical treatment and research	9 (47.4)
Laser treatments provided by:	
Periodontics faculty	1 (5.3)
Periodontics residents	4 (21.1)
Both periodontics faculty and residents	12 (63.2)
Periodontics faculty with resident assisting	2 (10.5)

* Each of the 19 programs using lasers responded to this multiple-choice question wherein they could choose more than one type of laser.

**Table 2:** Types of treatment provided with lasers in periodontics departments in the U.S. and Canada.

Types of Treatment Using Lasers	Number (%)
Periodontitis treatment	11 (57.9)
Periimplantitis treatment	16 (84.2)
Gingivectomy	13 (68.4)
Frenectomy	15 (78.9)
Canine exposure	7 (36.8)
Crown lengthening	5 (26.3)
Biopsies	8 (42.1)
Biostimulation (intraoral)	6 (31.6)
Biostimulation (extraoral)	5 (26.3)
Implant uncovering	4 (21.1)
Socket preservation	1 (5.3)
Other:	
Coagulation; bone removal; Leukopl	1 (5.3)

**Table 3:** Laser training in periodontics departments in the U.S. and Canada.

Laser Training Procedures	Number (%)
Type of Laser Training:	
Didactic only	2 (9.1)
Clinical only	2 (9.1)
Didactic and Clinical	15 (68.2)
No laser training	3 (13.6)
Is didactic course specific to laser training?	
Yes	9 (60.0)
No	6 (40.0)
Contact hours of didactic laser training:	
1–3 h	2 (13.3)
4–6 h	6 (40.0)
7–12 h	1 (6.7)
Over 12 h	6 (40.0)
Number of clinical laser procedures (CLP) performed by residents during residency:	
0 CLP	1 (5.9)
1–3 CLP	3 (17.7)
4–10 CLP	9 (52.9)
11–19 CLP	2 (11.8)
Over 20 CLP	2 (11.8)
Number of faculty members providing laser training in periodontics department:	
1 Faculty member	7 (63.6)
2 Faculty members	4 (36.4)

**Table 4:** Laser certification protocols followed in graduate periodontics programs in the U.S. and Canada.

Laser Certification Protocol	Number (%)
Minimum number of clinical cases completed for certification:	
1–4 clinical cases	2 (40.0)
5–9 clinical cases	2 (40.0)
10–15 clinical cases	1 (20.0)
Certification given to:	
All residents receive certification	4 (23.5)
Some residents receive certification based on certification program requirements	
Residents do not receive certification	1 (5.9)
N/A	9 (52.9)
	3 (17.7)

## Data Availability

Data is contained within the article or supplementary material.

## Supplementary Materials

The following supporting information can be downloaded at: https://www.mdpi.com/article/10.3390/dj10070119/s1. Table S1: Distribution of the types of lasers used by geographic location of the Dental Schools. Table S2: The DIODE Laser used for various periodontal procedures in the U.S. and Canada. Table S3: Distribution of the number of hours of didactic laser training in Graduate Periodontics Programs in the U.S. and Canada.
