# Supplementary material for: Status of North American Graduate Programs in Periodontics Providing Laser Education and Clinical Training: A Cross-Sectional Survey
Source: Dent J (Basel). 2022 Jul 1;10(7):119. doi: 10.3390/dj10070119 (PMC9317475; doi:10.3390/dj10070119)
Supplement: Supplementary file 1 [file dentistry-10-00119-s001.zip › dentistry-1753263-supplementary.pdf]

Table S1: Distribution of the types of lasers used by geographic location of the Dental Schools.

Type of Laser	Canada	Midwest US	Northeast US	Southeast US	Southwest US	West US	p-value
**CO2**							
Yes	---	---	4 (57.1)	2 (28.6)	1 (14.3)	---	0.1102
No	2 (16.7)	5 (41.7)	2 (16.7)	2 (16.7)	---	1 (8.33)	
**DIODE**							
Yes	2 (11.7)	4 (23.5)	5 (29.4)	4 (23.5)	1 (5.9)	1 (5.9)	0.7765
No	---	1 (50.0)	1 (50.0)	---	---	---	
**ERBIUM**							
Yes	1 (14.3)	1 (14.3)	5 (71.4)	---	---	---	0.0342*
No	1 (8.3)	4 (33.3)	1 (8.3)	4 (33.3)	1 (8.3)	1 (8.3)	
**NdYAG**							
Yes	---	3 (30.0)	3 (30.0)	2 (20.0)	1 (10.0)	1 (10.0)	0.2911
No	2 (22.2)	2 (22.2)	3 (33.3)	2 (22.2)	---	---	

Table S2: The DIODE Laser used for various periodontal procedures in US and Canada.

Laser Type	PERIODONTAL PROCEDURES (N=19)		p-value
	Yes n(%)	No n(%)	
**DIODE**	**BIOPSIES**		0.3216
Yes	8 (47.1)	9 (52.9)	
No	---	2 (100.0)	
**DIODE**	**BIOSTIMULATION**		0.5438
Yes	5 (29.4)	12 (70.6)	
No	1 (50.0)	1 (50.0)	
**DIODE**	**FRENECTOMY**		0.0351*
Yes	15 (88.2)	2 (11.8)	
No	---	2 (100.0)	
**DIODE**	**GINGIVECTOMY**		
Yes	13 (76.5)	4 (23.5)	
No	---	2 (100.0)	

			0.0877
DIODE	IMPLANT TREATMENTS		
Yes	12 (70.6)	5 (29.4)	
No	2 (100.0)	---	0.5322
DIODE	PERIODONTAL TREATMENTS		
Yes	16 (94.1)	1 (5.8)	
No	2 (100.0)	---	0.8947

*p<0.05

Table S3: Distribution of the number of hours of didactic laser training in Graduate Periodontics Programs in US and Canada.

No. of Hours of Didactic Training	Canada	Midwest US	Northeast US	Southeast US	Southwest US	West US
**0**	---	2 (100.0)	---	---	---	---
**1 – 3**	1 (25.0)	3 (75.0)	---	---	---	---
**4 – 6**	1 (16.7)	---	1 (16.7)	3 (50.0)	1 (16.7)	---
**7 – 12**	---	---	---	---	---	1 (100.0)
**Over 12**	---	---	5 (83.3)	1 (16.67)	---	---
